# Impact of errors in determining the start of myocardial enhancement during quantitative first pass perfusion cardiovascular magnetic resonance imaging

**DOI:** 10.1186/1532-429X-16-S1-P367

**Published:** 2014-01-16

**Authors:** Allison D Ta, Li-Yueh Hsu, Christopher A Miller, Hannah Conn, Susanne Winkler, Peter Kellman, Kim-Lien Nguyen, Sujata M Shanbhag

**Affiliations:** 1 USA; 2 Duke University School of Medicine, Durham, North Carolina, USA

## Background

Several studies have shown the potential of semi-quantitative and fully-quantitative analysis of cardiovascular magnetic resonance (CMR) perfusion images in the diagnosis of coronary artery disease (CAD). However, estimates of myocardial blood flow (MBF) rely on accurate selection of important timing parameters. In this study, we evaluate how the identification of the timing of the Start of Myocardial Enhancement (SME) affects MBF quantification. We hypothesize that incorrectly selecting the SME can adversely affect quantification of the MBF.

## Methods

20 patients, including 10 with significant CAD (defined as ≥70% stenosis of a major epicardial artery on invasive coronary angiography), underwent regadenoson stress perfusion imaging using a steady-state free precession dual sequence technique followed by rest perfusion imaging. Motion corrected CMR perfusion images were quantified using model constrained deconvolution to obtain MBF estimates (in mL/min/g). Manual selection of SME was used as a reference start time. To evaluate how the timing of SME affects MBF, we systematically and intentionally shifted SME before and after the reference start time (-2, -1.5, -1, -0.5, 0.5, 1, 1.5, 2 second offsets were evaluated). A normal and a stenotic sector were identified from the myocardial signal intensity curves for each study with significant CAD.

## Results

**Figure 1:**
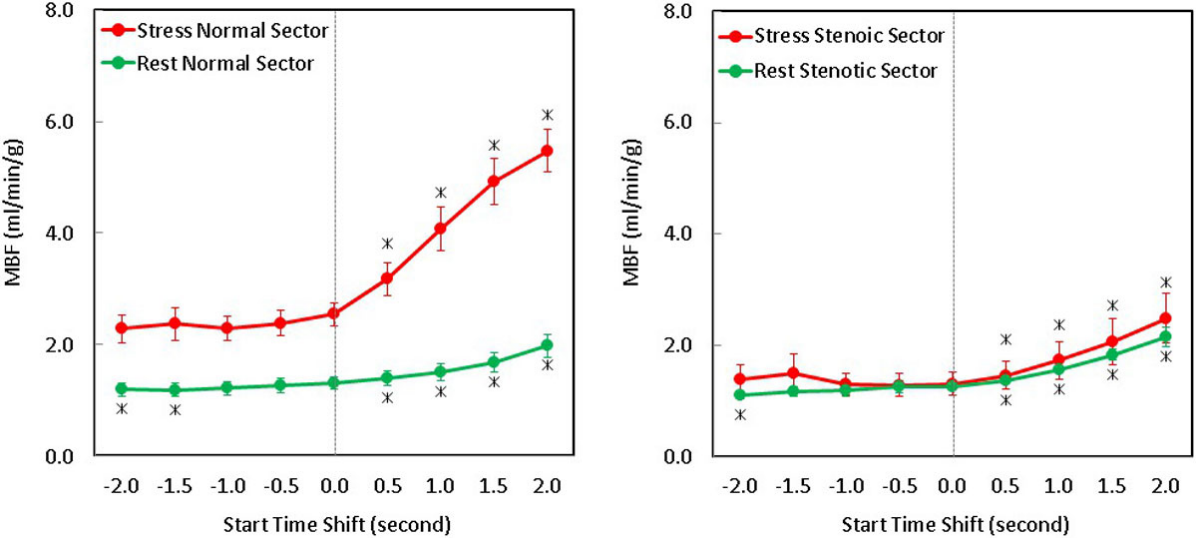
**Improper selection of Start of Myocardial Enhancement (SME) can lead to significant errors in Myocardial Blood Flow (MBF) quantification**. The average MBF is plotted against the time shift before and after the reference start time. MBF is minimally affected by shifting the SME earlier than the reference. However, when the SME is shifted after the reference, the MBF was significantly overestimated. The * represents MBF that is significantly (p < 0.05) different from the reference MBF.

A SME ≥1 second later than the reference SME resulted in a significant overestimation of MBF (p < 0.001), with mean MBF ≥25% than with the reference SME except in the normal sector for rest. The later SME was shifted, the more MBF was overestimated. Delaying SME by 2 seconds led to MBF estimates up to 3 times higher than when the reference SME was used (Figure 1). In both normal (Figure 1) and stenotic sectors, a SME 0.5 second after the reference resulted in significantly higher (p < 0.001) stress and rest MBF values. However, a SME 1.0 second or more before the reference SME didn't result in significant change of MBF estimates in both stress and rest studies (p = NS for both normal and stenotic sectors).

## Conclusions

This study demonstrates the importance of user-selected details when quantifying first pass myocardial perfusion. Selecting a timing point for SME that is the equivalent of one or two heart beats later than a reference standard results in a substantial overestimation of MBF, likely related to truncation of the myocardial time-intensity data. Truncation leads to overestimation of the initial contrast enhancement upslope particularly during stress. First pass CMR perfusion quantification should ensure that the onset of myocardial enhancement is properly detected.

## Funding

ADT is supported by the Sarnoff Cardiovascular Research Foundation. CM is supported by the National Institutes of Health Research, UK. ADT, LH, HC, SW, KN, PK, SS, MC, PB, and AA are supported by the National Institutes of Health, USA.

